# Marriage and Morals

**Published:** 1888-09-08

**Authors:** 


### The Doctor's Pulpit.

### MARRIAGE AND MORALS.

There is an intimate relationship between morals and brains. An immoral person is generally of defective intellect. The broad principles of sound morality are so evidently based on human nature and on the function of man in the world, that he who either fails to justly value them in his intellectual bushel measure, or, valuing them correctly, does not put them in practice, is to a certain extent a fool. This may be considered very plain speaking, and no doubt, measured by the standard of an undiscriminating plausibility, it is so. But the object of language, we submit, is the same as that of mathematics, viz., to express truth with the utmost possible accuracy. There would be no difficulty whatever in constructing a logical argument which should demonstrate infallibly the truth of the proposition that "the immoral man is a man of defective intellect"—in other words "a fool."

Conversely, morality means mind. The moral man, that is the man of voluntary morality, is a man of mind. The highest attainable elevation in morals is only possible to a man of the highest possible intellectual quality. This fact is in accordance with the teaching of all noble religions; but it is not exclusively the property of any of them. Philosophers ancient and modern, Greek and Roman, Agnostic and Theistic, are all agreed that morals and intellect are inseparable.

There are certain social usages which have a distinct influence on morality. Some of these usages tend to its conservation and healthy development; others tend to its destruction, and to the consequent enfeeblement and injury of the general intellect. It is but a commonplace to add that every worthy citizen feels bound to take note of the influence of all social usages; to maintain and defend those that make for the public weal; and to oppose and, if possible, exterminate those that are distinctly injurious.

Marriage is a social usage of the most widely prevalent character; it is so general as to be all but universal. It varies in the degree and limits of its strictness, but for our present purpose we shall confine ourselves to the consideration of ordinary monogamous marriage. What is the effect of this social usage on the general morality, and more remotely, though not less surely, on the general well-being of the people?

In this country, and as we know it, marriage is ordinarily preceded by love and courtship. The effect of that peculiar temper of mind expressed by the word "love," on both men and women, is very marked and profound. Poets have mostly agreed to declare that love is blind. That we do not believe. Love sees well enough the peculiarities and defects of its object. But it is itself of so large, generous, and forgiving a nature that it overlooks them. This power of large generosity and forgiveness is an intellectual quality of the first order; and if it could by any means be made universal among men, would do more to promote social amelioration and state progress than any of the panaceas which enthusiastic reformers have yet tried. As selfishness is at the root of almost all disorder and misery, so love is the beginning of all that is just and humane. This all-powerful feeling and temper of mind the young man and young woman who are genuinely in love with each other have a clear experience of for a longer or shorter time. The period of courtship is generally a period of moral development on sound lines. In this country it is the natural preliminary to marriage, and in this circumstance we are decidedly in advance, intellectually and morally, of at least one other leading nation.

But does not marriage, when fairly entered upon, terminate the love; and is there not then a descent from the high generosity and forgiveness of temper which prevailed in courtship? No! With those men and women who are naturally and incurably mean and base a more or less futile imitation of love may be followed in married life by a strong development of selfishness on both sides. But this is an abnormal case, it is not the rule. In dealing with broad questions the exceptions must not be allowed to obscure or invalidate the rule. Marriage, following upon ardent love, may for a time be a period of perfect happiness, and then there may follow a stage of mutual surprise and disappointment. But the stage of fuller knowledge and consequent disappointment once over, the really wholesome-natured man and wife settle down to mutual respect and forbearance, to a sober love which is as enduring as life, and compared with which the feelings characteristic of the courtship period were as the peach-bloom is to the peach.

Home and family life are the accompaniments, and indeed the fruit and essence of marriage. Home is the kingdom of the individual man and woman; and in a sense makes their completeness. An unmarried man is a wanderer, and mostly a failure. A married man who has been successful in obtaining and maintaining a reasonably happy home can never be called a complete failure. He may have set out for a bishopric and landed in a country vicarage; he may have aimed at the woolsack and alighted in a county-court judgeship; he may have intended to be a Wellington and ended by being a major on half-pay; but if he has a home worthy of the name, and wife and children whose loss he would feel to be irreparable, he is a successful man on the whole.

But is he not also a moral man? His pleasures lie at home, his wishes at home. This marriage relation, these family ties, these household gods, do they not build a wall about him which no wild beast of temptation can leap over? His home is his castle in a thousand senses; it is to him an unwritten code of morals, a sacred enclosure. He defends it, and it defends him. His mind is in repose. He can devote himself with whole-souled completeness to every duty which life may bring. Compare him with the unanchored man, beating about on life's ocean, a prey to a thousand temptations, to a thousand perils. He it is, and not the other, whose morals too often may not be inquired into, whose barque of character and prospects not seldom founders and goes down.

Monogamous marriage is the beginning of virtuous home-life; and virtuous home-life is the seed and germ from which public virtues spring. This is a proposition which needs no arguing; it is patent to general experience. Public and private virtue every man is interested in preserving. This is the atmosphere in which we all desire our sons and daughters to live. Can we contemplate for a moment those sons, and still less those daughters, entering upon a relation of mere convenience, forming a home which at any moment may be broken up, with the result of parents and children scattered far and wide, in mutual hatred, distrust, and dread? The home, we repeat, is the nursery of private virtue, and private virtue is the necessary basis and beginning of all morality. A people must be moral in order to be great, and in order to secure their own safety, permanence, and growth. Every man and every woman who has the mental capacity to understand these things will resolve, at all hazards, to conserve and make permanent the sacredness of the marriage relation.

